# Factors affecting recruitment and retention of community health workers in a newborn care intervention in Bangladesh

**DOI:** 10.1186/1478-4491-8-12

**Published:** 2010-05-03

**Authors:** Syed Moshfiqur Rahman, Nabeel Ashraf Ali, Larissa Jennings, M Habibur R Seraji, Ishtiaq Mannan, Rasheduzzaman Shah, Arif Billah Al-Mahmud, Sanwarul Bari, Daniel Hossain, Milan Krishna Das, Abdullah H Baqui, Shams El Arifeen, Peter J Winch

**Affiliations:** 1 International Centre for Diarrhoeal Disease Research, Bangladesh, Mohakhali, Dhaka, Bangladesh; 2 Department of International Health, Johns Hopkins Bloomberg School of Public Health, Baltimore, Maryland USA

## Abstract

**Background:**

Well-trained and highly motivated community health workers (CHWs) are critical for delivery of many community-based newborn care interventions. High rates of CHW attrition undermine programme effectiveness and potential for implementation at scale. We investigated reasons for high rates of CHW attrition in Sylhet District in north-eastern Bangladesh.

**Methods:**

Sixty-nine semi-structured questionnaires were administered to CHWs currently working with the project, as well as to those who had left. Process documentation was also carried out to identify project strengths and weaknesses, which included in-depth interviews, focus group discussions, review of project records (i.e. recruitment and resignation), and informal discussion with key project personnel.

**Results:**

Motivation for becoming a CHW appeared to stem primarily from the desire for self-development, to improve community health, and for utilization of free time. The most common factors cited for continuing as a CHW were financial incentive, feeling needed by the community, and the value of the CHW position in securing future career advancement. Factors contributing to attrition included heavy workload, night visits, working outside of one's home area, familial opposition and dissatisfaction with pay.

**Conclusions:**

The framework presented illustrates the decision making process women go through when deciding to become, or continue as, a CHW. Factors such as job satisfaction, community valuation of CHW work, and fulfilment of pre-hire expectations all need to be addressed systematically by programs to reduce rates of CHW attrition.

## Background

Community Health Workers (CHWs) can increase access to, and use of, health services, and have played a part in primary health care, tuberculosis, immunization and family planning programmes. CHWs received less attention in the 1990s, but now again are at the centre of discussions about how to improve coverage and equity, particularly in populations with limited access to health facilities [1]. With appropriate expectations and sufficient investment and support, CHWs have the potential to play an important role in strengthening weak health systems [2].

CHWs have been promoted for implementation of packages of interventions to reduce neonatal mortality such as antenatal home visits, promotion of immediate and exclusive breastfeeding, skin-to-skin care, appropriate care of the skin and umbilical stump [3-6], and recognition and treatment with antibiotics of sick newborns [7-10]. Delivery of interventions in the home by CHWs is viewed as critical during the first month of life, when many families observe a period of postpartum confinement which makes them less likely to seek care or advice from outside the home [11].

Syed and colleagues found that CHWs were effective in tracking pregnant women through the postnatal period and in raising awareness of appropriate maternal and newborn care practices [12]. Implementation of newborn care interventions is relatively complex compared to CHW-based interventions, such as the mass treatment of endemic diseases and the promotion of preventive services such as immunizations. To be effective, CHWs must gain mastery of a range of information and skills related to maternal and newborn care, and know how to adapt counselling strategies to households with varied composition and needs [3]. This, in turn, requires greater investment by programmes in CHW selection and training.

The term 'community health workers' can refer to a variety of health care providers such as village health workers, community resource people, traditional birth attendants or workers known by local names. While these providers are trained, they typically do not have any professional certification [1]. CHWs can deliver a variety of community-based health care services, and are particularly important in areas where the use of facility-based services is low.

Haines and colleagues propose four determinants of the success of a CHW programme: 1) national socioeconomic and political factors, including corruption and political will; 2) community factors such as location and infrastructure and health beliefs; 3) health system factors such as remuneration and supervision, and 4) international factors including migration flow and technical assistance [1]. CHWs require supportive supervision, clearly defined roles with specific tasks, locally relevant incentive systems that combine monetary and non-monetary incentives, recognition, training opportunities, community and policy support, and strong leadership [13,1]. All of these factors can play a role in the length of time a worker serves as a CHW.

In addition to the factors listed above, individual CHW motivation impacts retention and attrition. Motivation is driven by many elements including intrinsic factors such as an individual's work-related goals, as well as his/her sense of altruism, self-efficacy, and organizational commitment. Extrinsic factors include peer approval, the incentives provided, and the expectation of future paid employment [14-18]. These are similar to the factors found to affect motivation and retention of formally trained health workers in low income countries in a recent review [19].

Significant rates of attrition undermine programmes' investments in CHWs, and potentially limit the effectiveness of community-based interventions aimed at reducing neonatal mortality. Higher attrition rates are associated with volunteers [14]. One review reported that CHWs dependent on community financing are two times more likely to leave their posts than health workers compensated by government salaries [20]. A study in Bangladesh found reasons cited by CHWs for leaving their posts included lack of time to attend to their own children and other responsibilities, insufficient profit/salary, and their families' disapproval [21]. Another study in Nigeria found that village health workers stopped working because of low salaries, a lack of opportunity for advancement, a lack of credibility with the villagers, and poor supervision [22].

Henderson and Tulloch identified a number of key recommendations for retaining salaried health-workers [23]. Recommendations such as "improved working and living conditions," "improved supervision and management," clarifying "job descriptions, criteria for promotion, and career progression," "increasing education, training and professional development opportunities" and "social recognition" are applicable to both paid and volunteer workers [23]. Conversely, recommendations such as "strategies for return migration," "bonding and mandatory service" and "payment systems" are not relevant to CHWs [23]. While the recommendation of increasing salaries is not applicable to CHWs, increasing "benefits and allowances," as well as "performance-based non-financial incentives," of CHWs could increase retention [23]. Other studies suggest that strong social networks and social cohesion are important factors for CHW retention, and that CHWs benefitting from strong support system at the community level that validates their work and their role are more likely to continue in that role despite other potentially negative factors [24].

A newborn care intervention trial ("Projahnmo-1"), conducted in Sylhet District in north-eastern Bangladesh, evaluated the effectiveness of two different service delivery models of a package of maternal and newborn care interventions [25,26,9,10]. CHWs were the cornerstone of one of the community-based delivery strategies implemented in the Home Care intervention arm. In this arm, CHWs were the first level of health-workers engaged in service provision, serving a population of 4000, which is approximately 800 households. For pregnancy surveillance, each CHW spent on average two hours to cover 20 households. This was intended to replicate the coverage area of a similar cadre of governmental health workers. CHWs worked at the household and family level to promote Birth and Neonatal Care Preparedness. Additionally, they ensured the provision of safe delivery care, as well as essential newborn care during and after birth, by maintaining active coordination with the traditional and/or family birth attendants and the individual identified by the family to care for the newborn immediately following birth. The role of the CHW in this trial was involved and often complicated. Participating CHWs not only needed to be skilled technically and adept in clinical assessment, but they also were required to develop superior counselling skills - which were arguably equally important to their clinical skills.

CHWs were offered a remuneration package of 3200 Bangladeshi taka per month, which is equivalent to US$ 45 dollars. They were expected to work from eight in the morning to four in the afternoon six days a week, with newborn care visits to be made within the first day of life, even if that meant visiting the household on a holiday. Their work also involved paying informal unscheduled visits to households when and if families needed their assistance, especially when attending a sick neonate. The remuneration package did not include a scheduled incremental increase. Therefore, though it was comparable to that of other similar governmental job opportunities in the beginning (i.e. working as a Family Welfare Assistant or FWA), after a year there was a marked difference between their salary and that of the government FWAs.

In implementation of the Home Care intervention arm, CHW attrition was identified early on as a significant constraint on the effectiveness of the intervention package. This paper explores the causes of attrition, as well as how CHW attrition was analyzed and addressed by this community-based newborn care intervention in rural Bangladesh.

## Methods

Sylhet is known to be a relatively conservative region of Bangladesh. Recruiting women for a CHW position, a type of work unfamiliar to the community, proved to be difficult at first. Initially the Sylhet project planned to recruit married women with 10 or more years of education to serve as CHWs. The educational requirement was necessary because of the detailed record forms CHWs had to complete as part of the study. Due to the shortage of married women with the requisite educational background, along with the initial reluctance of women to serve as CHWs in general, the project ultimately recruited single women.

A total of 41 CHWs were recruited at the beginning of the intervention in Sylhet District who fulfilled the following criteria:

1) female,

2) local resident in the area of assignment,

3) preferably married (this criterion was dropped when it proved impossible to identify sufficient numbers of eligible married women),

4) aged between 20 and 40, and

5) secondary school leaving certificate (SSC pass).

Initially there were 38 CHW service areas (areas covered by one CHW), thus three CHWs were available to serve as replacements in the event of attrition among the other 38 CHWs. In the early months of the project, two service areas were deemed too large for effective coverage by 1 CHW, and were subsequently divided, ultimately resulting in 40 CHW service areas.

CHWs were recruited through advertisements placed in a local newspaper. Candidates meeting the criteria sat for a written general knowledge examination that covered questions such as: "What are the main child health problems in Bangladesh?" and "What is an NGO?". Candidates who passed the written examination were interviewed at the project office. If they passed the interview and agreed to work they then received six weeks of training. After training they were evaluated on maternal and newborn care knowledge and relevant skills for intervention.

Initial CHW responsibilities included visiting assigned households to identify pregnant women (pregnancy surveillance). Subsequently, CHWs began providing registered pregnant women with birth preparedness messages and materials. Attending deliveries, attending sick newborns, referral of women and newborns to care, and, in the case of referral failure, treatment of sick newborns in the home with injectable antibiotics [10], as well as filling out forms, were other tasks added over time.

Over the course of the four-year project, a total of 73 CHWs were recruited either initially (41 CHWs) or later on to replace CHWs who left the project, requiring additional efforts by the programme to recruit and train replacement CHWs. The total period of intervention was 36 months. Thirty-two CHWs (referred to as former CHWs in this paper) left the project during this period, of whom 15 left within one year, and another 10 by the end of the second year of the project. Training of the replacement CHWs had a higher unit cost, because they were trained on an on-going basis, sometimes only a few at a time. Dedicated trainers were initially recruited for the project only on a short-term basis, thus the responsibility for training new CHWs fell to the supervisors in the later phase of the project. This proved extremely difficult, given their routine programme responsibilities. The project took the following steps in order to address the problem:

• Project staff, including senior level managers, visited the houses of CHWs and talked with their parents and guardians, communicating the aims and activities of the programme and emphasizing the benefits to the community as well as to their daughter/s.

• From the outset of the project, staff of the implementing non-governmental organization partner (Shimantik) held community advocacy meetings to explain the project and respond to community concerns. The NGO added a new step of initiating dialogue with the parents and guardians of the new CHWs at the time of recruitment in order to explain the project and roles of the staff members.

• A number of field-level workers were given the opportunity to become supervisors based on exemplary performance.

• A number of incentives were created, such as incentives for the CHWs to attend deliveries at night.

Data from three different sources are presented. The first source is employment records of CHWs in the newborn care intervention trial in Sylhet District [9], with mention of differences with another trial in Tangail District, Mirzapur Subdistrict (Table 1) [27].

**Table 1:** CHW demographic characteristics and patterns of recruitment and attrition in Sylhet District, Bangladesh

Demographic characteristics	
Total CHWs employed over life of project	73 CHWs

Mean age of CHWs (Standard deviation)	23.3 (4.3)

	Frequency (Percent)

Marital status at time of recruitment	
Unmarried	45 (61.6)
Married	19 (26.0)
Divorced/separated	9 (12.3)

Years of education at time of recruitment	
9 years	1 (1.4)
10 years	54 (74.0)
12 years	17 (23.3)
14 or more years	1 (1.4)

**Patterns of recruitment and attrition**	

Number of CHW service areas (zone covered by 1 CHW)	40 areas
Service areas with same CHW from start to end	21 areas (52.5)
Service areas with CHW attrition during project	19 areas (47.7)

CHW attrition during the project	
No attrition: CHW worked until end of project	40 (54.8)
Attrition initiated by CHW and/or family	26 (35.6)
Attrition initiated by project	7 (9.6)

Reasons for attrition initiated by CHW or family	
	
**CHW left for family reasons**	14
Marriage	11
Family opposed to her working as CHW	3
Husband got work in Dhaka	0
	
**CHW left for work-related reasons**	11
Left to take other position	6
Workload considered too heavy	2
Wanted promotion but not granted	2
Wanted to change to other service area	1
	
**CHW left to pursue higher education**	1

**Reasons for attrition initiated by project**	
Promoted to higher position with project	4
Terminated due to poor performance	3

Sample: All 73 current and former CHWs in the project, working in 40 CHW service areas.

The second source of data is a survey of factors affecting retention and attrition administered to 69 of the 73 current and former CHWs in Sylhet District. Semi-structured questionnaires were administered in 2005 to CHWs currently employed and those who left the job in order to elicit information on job satisfaction, demands, and aspirations. This was a self-administered questionnaire in which, after a brief explanation of how the questionnaire was arranged, respondents were asked to complete it based on their interpretation. The self-administered questionnaire for CHWs had 45 questions, divided into four parts: 1) Personal and family history, 2) Motivation behind CHW work, 3) Experiences related to CHW work, and 4) Reasons for leaving. There were both multiple-choice and open-ended questions. A mid-level manager who was not directly supervising the CHWs was chosen as the facilitator for the self-administered questionnaires. The respondents were all CHWs, both those who had left and those still working for the project. CHWs were given complete freedom to respond as they saw fit. CHWs were given the confidence and assurance that their names would be removed at the time of analysis of the data. Data were further strengthened by informal discussions with field managers and supervisors aimed at gaining insight from their field experiences. Finally, data are included from a complementary qualitative process documentation exercise consisting of in-depth interviews, focus group discussions, a review of project records (i.e. recruitment and resignation), and informal discussion with key project personnel.

## Results

### Demographic characteristics of CHWs in Sylhet

Table 1 displays CHW demographic characteristics and patterns of recruitment and retention in Sylhet District. Compared to CHWs in a similar project in Tangail District in central Bangladesh, CHWs in Sylhet were significantly younger (mean age 26.8 years in Tangail versus 23.3 years in Sylhet, p < 0.001) and much more likely to be unmarried (25.0% in Tangail versus 61.6% in Sylhet, p < 0.001).

### Recruitment and retention of CHWs

Rates of CHW attrition were far higher in Sylhet than in Tangail over the 36-month period of the two projects (2004-2006). In Sylhet 52.5% of the 40 CHW service areas had the same CHW from the beginning to end of the project (Table 1), compared to 69.4% of the 36 CHW service areas in Tangail. A total of 73 CHWs were employed over the life of the project in Sylhet. The primary reasons for CHW attrition are grouped into 4 categories: family reasons, work-related reasons, education opportunities, and actions taken by the project. Of the four categories, family-related reasons are the most important, notably opposition by families to daughters working as CHWs, and CHWs ceasing to work after marriage. Six CHWs left when they obtained government positions, most notably work as primary school teachers (Table 1). Four CHWs were promoted to supervisory positions, and three were fired due to poor performance (Table 1). Promotion was one strategy adopted by the project to retain the best CHWs. There were no significant associations with age, marital status and education between CHWs who left the project and those who continued until the end. This is partly to be expected, because the selection criteria for CHWs resulted in a group of CHWs being selected that had little variation in these variables.

### Factors influencing decision to become a CHW

Tables 2, 3 and 4 present data from the survey of 69 of the 73 current and former CHWs conducted at the Sylhet site in 2005. This survey excludes the four replacement CHWs hired subsequently. Table 2 displays factors influencing the decision to become a CHW. The two groupings of factors most commonly cited in Table 2 are self-development (desire to improve skills) and the desire to improve community health. Other factors which motivate women to serve as CHWs are: the desire to use available time productively and, less commonly, value and recognition from the community and aspirations for financial independence. Most differences were non-significant, in part due to limited statistical power. Former CHWs were significantly more likely to say that family and friends encouraged them to apply for the job (p = 0.022), that they knew much time would be required, but they had a lot to offer (p = 0.024), and that they wanted to learn about maternal and newborn care (p = 0.039).

**Table 2:** CHW survey in Sylhet District, Bangladesh: factors influencing decision to become a CHW

		Current N = 46	Former N = 23	Total N = 69	p from Chi-square
Self-development	I expected the experience will enhance my communication skills.	38 (83%)	17 (74%)	55 (80%)	N.S.
	
	I expected to get involved in the community.	33 (72%)	15 (65%)	48 (70%)	N.S.
	
	I expected I will have a new sense of self-pride and accomplishment.	37 (80%)	18 (78%)	55 (80%)	N.S.
	
	I hoped to gain skills that would enable me to work as a health practitioner.	34 (74%)	20 (87%)	54 (78%)	N.S.
	
	I wanted to eventually work elsewhere and knew that field experience was required.	28 (61%)	13 (57%)	41 (59%)	N.S.

Financial independence	I needed to earn money to help support my family.	23 (50%)	8 (35%)	31 (45%)	N.S.
	
	I wanted to earn money to save for school	5 (11%)	2 (9%)	7 (10%)	N.S.

Value and recognition	My family and friends encouraged me apply for the job.	16 (35%)	13 (57%)	29 (42%)	0.022
	
	I knew of other CHWs who were respected in the community.	12 (26%)	4 (17%)	16 (23%)	N.S.
	
	I believed working as a CHW was a respectable, honourable job.	29 (63%)	12 (52%)	41 (59%)	N.S.

Use of available time	I wanted to spend my time constructively.	23 (50%)	11 (48%)	34 (49%)	N.S.
	
	I knew much of my time would be required, but I had a lot time to offer.	20 (43%)	16 (70%)	36 (52%)	0.024

Improve community health	I wanted to learn about newborn and maternal care for my own family.	29 (63%)	20 (87%)	49 (71%)	0.039
	
	I wanted to improve the health of the community.	36 (78%)	22 (96%)	58 (84%)	N.S.

Sample: 69 of the 73 current and former CHWs in the project at time of the survey

**Table 3:** CHW survey in Sylhet District, Bangladesh: reasons given by current CHWs for continuing to work

		Percent (%) of CHWs agreeing
Recognition	I feel the people in the community need me	37(86%)

Financial and time allowances	I need the money/salary.	41 (95%)
	
	I have time because I am not married.	15 (35%)

Job satisfaction	My work is enjoyable.	30 (70%)
	
	I like travelling in the community.	25 (58%)

Support	Other CHWs have encouraged me to stay.	18(42%)
	
	My family has encouraged me to stay.	28 (65%)
	
	My supervisor has encouraged me to stay.	28 (65%)
	
	I have someone to help me with my duties at home.	23 (53%)

Personal development	Working as a CHW will help me get a better job.	37 (86%)
	
	I applied for more education or another job, but have not/will not receive it.	4 (9%)

Sample: 43 of 46 current CHWs in the project at the time of the survey

**Table 4:** CHW survey in Sylhet District, Bangladesh: job satisfaction and challenges related to work among current and former CHWs

		Current N = 46	Former N = 23	Total N = 69	p from Chi-square
**Fulfilment of pre-hire expectations**					

Self-development	I have enhanced my communication skills.	38 (83%)	18 (78%)	56 (81%)	N.S.
	
	I have enjoyed working in the community.	27 (59%)	12 (52%)	39 (57%)	N.S.
	
	I have a new sense of self-pride and accomplishment from my job.	35 (76%)	18 (78%)	53 (77%)	N.S.
	
	I feel that I have adequate field experience to qualify for other public health jobs.	32 (70%)	15 (65%)	47 (68%)	N.S.

Dissatisfaction with pay	I think I should be paid more.	39 (85%)	20 (87%)	59 (86%)	N.S.
	
	I do not have enough money to cover my work-related expenses.	22 (48%)	13 (57%)	35 (51%)	N.S.

Recognition and contribution to community health	Newborn family valued my night attendance to delivery or newborn illness.	29 (91%)^a^	7(88%)^a^	36 (90%)^a^	N.S.
	
	Newborn family valued my care even in the event of a neonatal death	27 (90%)^b^	7 (88%)^b^	34 (89%)^b^	N.S.
	
	I am proud to be a CHW and feel that my work is valued.	32 (70%)	14 (61%)	46 (67%)	N.S.

Use of time	The job has required the amount of time than I expected it would.	27 (59%)	14 (61%)	41 (59%)	N.S.

**Challenges in serving as CHW**					

Difficult aspects of work	Conducted night visits for delivery or illness	32 (70%)	8 (35%)	40 (58%)	0.0058
	
	Experienced death of newborn under her care	30 (65%)	8 (35%)	38 (55%)	0.017
	
	Working outside home community or area	11 (24%)	7 (30%)	18 (26%)	N.S.

Familial reaction to night work or work outside home area	My family discouraged and/or questioned necessity of my conducting night visits.	13 (41%)^a^	5 (63%)^a^	18 (45%)^a^	N.S.
	
	Moving outside home area a problem since I am not living with other family members.	6 (55%)^c^	3 (43%)^c^	9 (50%)^c^	N.S.
	
	I do not feel a part of the community.	4 (36%)^c^	3 (43%)^c^	7 (39%)^c^	N.S.

**Supervision, support and workload**					

Supervision & support^d^	I receive immediate help when I need it.	29 (63%)	17 (74%)	46 (67%)	N.S.
	
	I receive regular feedback from my supervisors on my performance and quality of work.	39 (85%)	22 (96%)	61 (88%)	N.S.
	
	I feel that if I have a concern, I can share it with my supervisor/manager.	38 (83%)	20 (87%)	58 (84%)	N.S.
	
	I feel that my supervisor understands my job and the challenges I face.	35 (76%)	16 (70%)	51 (74%)	N.S.

Workload	I have enough time to complete my daily tasks.	28 (61%)	15 (65%)	43 (62%)	N.S.
	
	I have enough time for my personal duties.	3 (7%)	2 (9%)	5 (7%)	N.S.
	
	My schedule is flexible, can tailor it to my preferences & those of families I work with.	22 (48%)	13 (57%)	35 (51%)	N.S.

Sample: 69 of the 73 current and former CHWs in the project at time of the survey

[a] Among CHWs who had conducted nights visits [Total = 40 (32 Current, 8 Former); [b] Among CHW who had experienced a neonatal death [Total = 38 (30 Current, 8 Former); [c] Among CHWs who worked outside of residential area [Total = 18, 11 Current, 7 Former] [d] Missing significant amount of data

### Reasons given by CHWs for continuing to work

Table 3 shows reasons given by current CHWs in the survey in Sylhet for continuing to work. The financial incentive was the biggest motivating factor (95%) for retention, though it did not rank among the top reasons the women chose to become CHWs. Also prominent reasons for continuing to work were the recognition derived from being needed by the community (86%) and the expectation of landing a better job due to the experience gained as a CHW (86%). Less frequently, CHWs mentioned enjoyment associated with the work, and support and encouragement by family and supervisors as motivating factors.

Data on CHW retention were also collected from a process documentation exercise carried out mid-way through the project at the Sylhet site. Respondents were asked why they became CHWs, and the majority explained that they wanted to help improve newborn and maternal health in their community. Beyond the benefits of employment, many CHWs anticipated that the position would positively impact their families and neighbours, as well as their own personal lives. For many of these women, "work is good; whatever it is. Joblessness is a curse." For most CHWs, this was their first job, and many considered it to be a gateway to future work. Two of the CHWs explained:

"Initially it was only money, but later I figured that this job may help me in the future to get better jobs. Maybe someday I will be able to become a paramedic."

"If I get another job, be it a government job or not, I would like it to be related to health, since I know a lot about health now."

The ability to make financial contributions to the family is a substantial impetus in CHW retention, despite concerns about the heavy workload and discomfort with the evening and holiday travel required by the job.

Many CHWs expressed interest in pursuing future employment as another type of health care provider and doing work such as delivering babies, becoming a paramedic, or working for the government as a community or facility-based health worker. A few hoped to work as a government school-teacher. Additional motivating factors included meeting other women with similar interests and goals. Several CHWs expressed desires of independence and the ability to help provide financially for their families: for their husbands and children (for those who were married) and for their siblings (for those who were unmarried). Despite attempts at saving money, CHWs reported difficulty especially in the few cases where CHWs were recruited outside their cluster areas and thus were required to pay rent by their jobs.

In-depth qualitative interviews suggest that CHWs would recommend CHW work to a relative or friend, as long as all the relevant aspects of the job were explained in advance. Women said that they would stress the importance of CHW work in reducing neonatal mortality in the community, the opportunity for the CHW to expand her knowledge of health-related matters, and the benefit of having a monthly salary. Two CHWs felt that the role of CHW increased women's independence and marketability for better-paying jobs, although concerns were raised about the response of the community and overall handling of the workload:

"I would recommend the CHW job, but I will let them know everything, including how hard it is. I would also warn them of problems with not being paid travel bills regularly. If she likes what I say, then she will go ahead and work."

### CHW job satisfaction and work-related challenges

Table 4 from the CHW survey displays responses to questions on the CHW survey in Sylhet District on job satisfaction and work-related challenges. Two challenges to carrying out the work are mentioned significantly more often by current CHWs than former CHWs: the necessity of making visits at night to attend deliveries (p = 0.0058), and experiencing the death of a newborn (p = 0.017). Other challenges commonly cited that do not differ significantly between current and former CHWs include dissatisfaction with pay, underestimation of required time for CHW-related work, working outside of one's home area; and familial opposition to CHW work. In in-depth interviews, former CHWs were more likely to report that the CHW job did not meet their expectations for self-development or value and recognition. They were also more likely to be dissatisfied with their remuneration and less likely to attribute CHW experiences to being recognized or valued by the community.

Most current and former CHWs noted in in-depth interviews that movement throughout all parts of the community was a problem, both for them and for their families. Some of the CHWs commented that they were initially very reluctant to move around and were referred to as 'girls' by community members. In the local Sylheti language, "phuri" meaning girl is used for any unmarried woman, rather than "mohila" meaning woman. The project CHWs were referred to as "phuri" because they were unmarried.

Current CHWs reported, however, that much of the initial discomfort and apprehension had dissipated since many of them began wearing a Muslim headdress (*niqab*). Now they feel more confident and move around alone, taking the bus or any other vehicle to travel to distant villages.

"Now we are known to the community; otherwise we wouldn't have been able to work. Some people are very helpful - they hail rickshaws, boats, or whatever is needed. They know we are CHWs and are responsible and alert. It is not bad now"

In in-depth interviews with four former CHWs, concern about moving around the community and familial disapproval were consistent themes. Some CHWs explained that their family members are anxious, especially when they return home late. Others say their families worry because they are young and vulnerable to danger. Many CHWs are even reluctant to discuss the complete responsibilities of their work with their families, noting, for example:

"They wonder what kind of job it is that requires women to stay out so long. If my brother was here in the country, then I wouldn't be able to work as a CHW."

"My father is the sufferer...He used to be the alternate imam of the village. Now half of the people do not want to stand behind him in the prayers. They say his daughters work for NGOs, which is not right for a religious person."

Despite the apparent antagonism and disapproval from families, most have accepted the CHW-work, as a result of the substantial contribution to the family. When asked why they had continued working as CHW, the majority explained that, at best, the job simply "pays". Others noted reasons for staying similar to their reasons for joining - an opportunity to educate the community on beneficial maternal and neonatal care practices, and general good feelings resulting from the ability to provide treatment for sick newborns.

Other reasons for CHW attrition included better job offers, lack of security with their present job, insufficient compensation given the breadth of work, discomfort moving in predominately male settings, and marriage duties. Many CHWs applied for schoolteacher and Family Welfare Assistant positions with the government. Government posts are highly attractive in Bangladesh, and CHWs frequently commented on their job's excessive workload compared with other the government positions.

Disapproval from families regarding household visits by male supervisors, problems with travel bills, and overall dissatisfaction with management styles were other factors cited. One CHW commented:

"Management used to go overboard - they didn't realize that the job wasn't everything in my life...We were looked at with suspicion whenever we talked to anyone outside the scope of work. If it was a guy, then things were simply unbearable, dirty."

CHWs who had left the project had difficulty suggesting what could have been done to influence their decisions to leave. Two women felt, regardless of efforts by management specifically to encourage them to stay, their families would not have agreed. Families' perceptions of the nature of the job made them uncomfortable with their daughters' position.

"I mentioned this [working as a CHW] to my cousin, but her father didn't let her work since he heard [the project] works for family planning."

## Discussion

Attrition of CHWs had serious implications for the effectiveness of the package of maternal and newborn care interventions being tested. High rates of attrition in the Sylhet site threatened the continuity of the project activities, both in the field and within the families of the pregnant women and newborn babies. High attrition potentially could adversely impact the project's credibility, due to irregular and inadequate interaction of the CHWs with the community. Attrition of CHWs put strains on project management, due to the intensive effort required for recruitment, training and supervision of new CHWs. This project focused heavily on clinical assessment of the newborn babies, so considerable effort was needed to ensure adequate training and supervision by personnel with appropriate clinical skills.

High rates of attrition increased the cost for training since the original plan was to train around 40 CHWs, while the project ended up having to train more than 70 - doubling the projected amount for this purpose. However, it is noteworthy that attrition is not all negative, and to some degree, is to be expected. Attrition indicates that some women were confident enough to seek opportunities beyond what their local communities offered and make choices that benefit themselves and their families. However, measures can and should be taken for the sake of the project to minimize its rate. The results of this study, coupled with the experiences in the current second phase of the Projahnmo project, indicate that newborn health programmes can proactively address many problems at the planning stage by ensuring appropriate levels of remuneration or putting in place alternative systems of incentives, and training supervisory personnel on how to identify and address causes of attrition.

**Figure 1:**
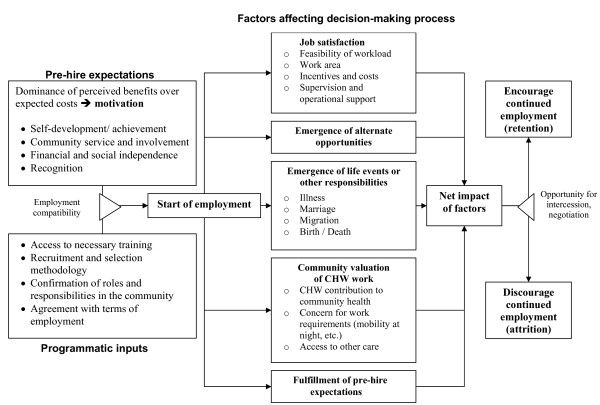
**Framework for decision-making process in retention and attrition of CHWs**.

Factors affecting retention and attrition overlap, but are not necessarily opposites. Decisions for retention or resignation are based on a complex set of trade-offs between different factors affecting different individuals differently. We need to understand how these factors interact and how the process is triggered. In this paper, we have proposed a general framework for the individual decision making process with regard to retention and attrition (see Figure 1).

Figure 1 shows the pre-hire expectations that motivate a woman to become a CHW, including how the work will contribute to her self-development, her ability to be involved with her community, her financial and social independence, and the community's recognition of her work. These motivating factors coupled with inputs by the programme, such as access to training and agreement with the terms and conditions of employment, determine whether she becomes a CHW.

Once employed, five key factors influence CHW retention. The first factor is job satisfaction, which includes issues such as the amount of work the CHW is expected to perform, how close her work is to her home, incentives and costs to being a CHW, as well as the kind of supervision and operational support she receives. Second is whether alternative job opportunities become available that are either more attractive or long-term, rather than project-based. The third factor is the occurrence of significant life events, such as marriage, childbirth, moving to another community, personal illness, or the occurrence of illness or death in the family. The fourth factor is the value the community attributes to CHW work, and the existence of other options for health care in the community. The final factor contributing to CHW retention is the extent to which her pre-hire expectations were realized. These bear similarities to factors identified in a recent review on motivation and retention in formally-trained workers [19].

In the Sylhet context, some of the actions that may produce positive results in terms of retaining CHWs could be:

• Salary needs to be comparable with other government and NGO positions with similar responsibilities. Also, salary needs to be fixed with the stated roles and responsibilities, with regular increments, bonuses, and other possible allowances provided as incentives. (factor # 1, 2)

• Hardship allowances can be provided to ensure newborn visits during holidays and beyond official work-hours. (factor # 1)

• Sick leaves can be provided for unforeseen sicknesses and medical emergencies. (factor # 1, 3)

• In response to families' concerns regarding their daughter's CHW responsibilities, group discussions can be arranged to engage the family in particular and the community as a whole to increase the sense of ownership of the project. This can be an effective community mobilization tool. (factor # 4)

• At the planning and training stage of a project, it should be ensured that the expectations of CHWs regarding their roles and responsibilities are clear and that they do not evolve into different roles and responsibilities over time. There should be frank discussions of less pleasant aspects of the job, such as late hours and holiday duties, so that they are fully aware of what to expect. (factor # 5)

## Conclusions

These five factors will impact each CHW differently. As she weighs the factors and their impact on her own life, she will decide whether the benefits of being a CHW outweigh the costs. The framework shows that at this point in the decision-making process, there is an opportunity for the CHW and the project to negotiate to increase the benefits of employment and/or reduce the costs to the CHW. Finally, the CHW will consider the five factors and the results of any negotiation with the project and determine whether she will continue to work as a CHW.

## Competing interests

The authors declare that they have no competing interests.

## Authors' contributions

All authors read and approved the final manuscript.
